# Successful CAR-T cell therapy in a refractory MCL patient with bacterial, fungal and COVID-19 infection: a case report

**DOI:** 10.3389/frtra.2023.1238494

**Published:** 2023-09-19

**Authors:** Vera Radici, Cinzia Giagulli, Eugenia Accorsi Buttini, Mirko Farina, Nicola Polverelli, Duilio Brugnoni, Marco Chiarini, Anna Galvagni, Camillo Almici, Emilio Ferrari, Andrea Bianchetti, Stefania Masneri, Alessandro Leoni, Federica Re, Simona Bernardi, Michele Malagola, Alessandro Re, Arnaldo Caruso, Domenico Russo

**Affiliations:** ^1^ Unit of Blood Diseases and Bone Marrow Transplantation, Cell Therapies and Hematology Research Program, Department of Clinical and Experimental Science, University of Brescia, ASST Spedali Civili di Brescia, Brescia, Italy; ^2^ Section of Microbiology, Department of Molecular and Translational Medicine, School of Medicine, University of Brescia, Brescia, Italy; ^3^ Flow Cytometry Unit, Clinical Chemistry Laboratory, ASST Spedali Civili di Brescia, Brescia, Italy; ^4^ Stem Cell Laboratory, Servizio di Immunoematologia e Medicina Trasfusionale, ASST Spedali Civili di Brescia, Brescia, Italy; ^5^ CREA Laboratory (Centro di Ricerca Emato-Oncologica AIL), ASST Spedali Civili di Brescia, Brescia, Italy; ^6^ Hematology Unit, ASST Spedali Civili di Brescia, Brescia, Italy

**Keywords:** COVID-19, infection, CAR-T cell, lymphoma, mantle cell lymphoma (MCL)

## Abstract

**Background:**

The COVID-19 pandemic has had a significant impact on the management and care of onco-hematological patients, particularly those with lymphoproliferative disorders who are at higher risk for COVID-19 associated bacterial and fungal superinfections.

**Case presentation:**

We present the successful treatment of a 44-year-old male patient with refractory mantle cell lymphoma treated with chimeric antigen receptor T (CAR-T) cell therapy, despite concurrent COVID-19 infection. The patient developed grade II cytokine release syndrome, requiring admission to the intensive care unit. The CAR-T cells expanded effectively, and the patient achieved complete metabolic remission. During the treatment course, the patient experienced complications including COVID-19-associated pulmonary aspergillosis and a co-infection with *Stenotrophomonas maltophilia* and the SARS-CoV-2 omicron variant. Prompt antifungal and antibacterial therapy, along with appropriate COVID-19 treatment, led to the resolution of these infections. Dexamethasone was also administered to reduce inflammation and aid hematologic recovery. Despite the presence of multiple infections, the patient achieved complete remission of lymphoma, highlighting the effectiveness of CAR-T cell therapy in this high-risk patient.

**Conclusion:**

Despite the challenges posed by concurrent infections, the decision to proceed with CAR-T cell therapy in this patient proved to be successful, resulting in complete remission of lymphoma. Early initiation of supportive therapies and the use of dexamethasone contributed to the resolution of complications. This case underscores the importance of individualized decision-making and the potential benefits of CAR-T cell therapy in similar high-risk patients.

## Introduction

The pandemic sustained by severe acute respiratory syndrome coronavirus 2 (SARS-CoV-2/COVID-19) had a dramatic impact on the management and care of onco-hematological patients (1). Due to the disease and therapy-related immunosuppression, patients with lymphoproliferative disorders are particularly exposed to increased morbidity and mortality for COVID-19 associated bacterial and fungal superinfections (2).

We report a case of a refractory mantle cell lymphoma (MCL) successfully treated with chimeric antigen receptor T (CAR-T) cell therapy while bacterial, fungal, and COVID-19 infections were occurring.

## Case presentation

A 44-year-old male patient was diagnosed in June 2021 with MCL, pleomorphic variant, stage IV B, high-risk mantle cell lymphoma international prognostic index (MIPI), without significant co-morbidities. As a first-line treatment, the patient received six cycles of alternating R-CHOP (rituximab, cyclophosphamide (CTX), doxorubicin, vincristine, and prednisone)/R-DHAP (rituximab, dexamethasone, high-dose cytarabine, and cisplatin). According to the Lugano criteria (3), he achieved only a partial response (PR) given the persistence of an abdominal mass (19 × 11 mm), documented by computed tomography (CT) scan.

In December 2021, ibrutinib was started, but the patient was urgently readmitted for massive hemoperitoneum 1 month later. Once the acute complication was solved, the patient started the first course of R-BAC (rituximab, bendamustine, cytarabine) resulting in a stable PR after the completion of three courses, in February 2022. One month later, a paucisymptomatic SARS-CoV-2 infection was documented, resulting in nasal swab negativization after Nirmatrelvir/Ritonavir treatment. Previously the patient had received three doses of the Pfizer vaccine. Genotyping was performed utilizing real-time PCR (RT-PCR) multiplex technique, unveiling the B.1.1.529 variant (referred to as omicron). Subsequently, sequencing of the S, N, and M genes was conducted using the Sanger method, disclosing the presence of the B.1.1.529 BA.5 lineage variant (see the Material and Methods section).

In May 2022, a further increase of mesenteric adenopathies indicated a novel disease progression. Vincristine (VCR, 2 mg) and corticosteroids (PDN, 1 mg/kg) were promptly administered with containment intent. In the meantime, the SARS-CoV-2 PCR test on nasal swab samples was alternating as positive and negative, while the patient always remained asymptomatic.

Considering the rapid progression of the disease and the COVID-19 asymptomatic infection, the patient was included in a CAR-T cell program. As bridging chemotherapy, the patient received high-dose cytarabine (ARA-C) followed by a short course of corticosteroids and CTX. In addition, a dose of Evusheld (tixagevimab 300 mg + cilgavimab 300 mg) was administered for an increased risk of progression to the severe form of COVID-19 and the susceptibility of this drug to the variant involved. Despite a further nasal swab positivity for COVID-19, the patient remained asymptomatic. The patient’s individual HEMATOTOX score before lymphodepleting chemotherapy was high, due to C-reactive protein >3 mg/dl and ferritin >2,000 ng/ml (4).

Due to his peculiar clinical condition, both lymphodepletion therapy (including fludarabine and cyclophosphamide) and Brexu-cel were administered in a negative-pressure room at the Department of Infectious Diseases on 12 July 2022. Although the nasal swab was positive for COVID-19 by RT-PCR, the patient continued to be persistently asymptomatic.

**Figure 1:**
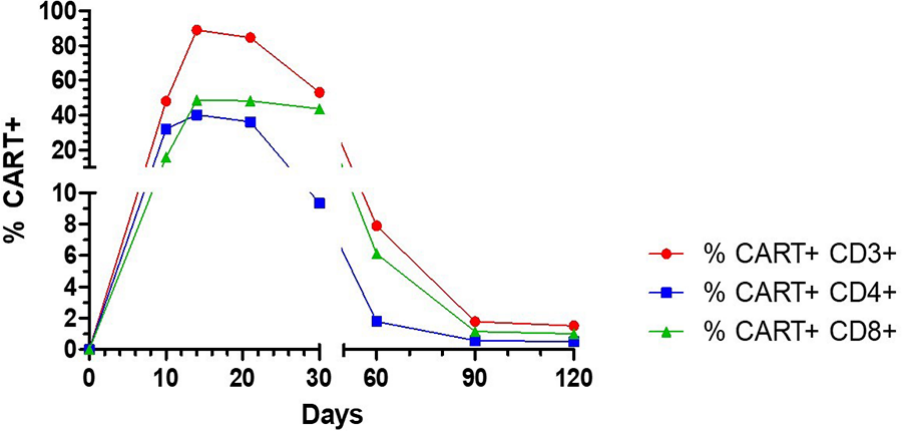
The percentage of CD3+ CAR+ T cells in total leucocytes, as analyzed by flow cytometry.

On day +6 from CAR-T infusion, the patient developed a grade I cytokine release syndrome (CRS), characterized by a fever of 38.7 °C in the absence of other symptoms. For the persistence of symptoms, three doses of Tocilizumab were administered every 8 h, and empirical antibiotic therapy with piperacillin-tazobactam was started. On day +9, due to a worsening of CRS (grade II) characterized by fever, hypotension, and desaturation, the patient was admitted to the intensive care unit (ICU) and treated with dexamethasone and low-flow oxygen therapy with an improvement of symptoms. On day +10, the circulating CAR-T cells were first checked, reaching a peak of 88% of total lymphocytes on day +14 (good expander) and then decreasing to 8% at day +60 (Figure 1). On day +21, a CT scan documented the radiological signs of possible fungal pneumonia. *Stenotrophomonas maltophilia* and *Aspergillus niger* were isolated on sputum. Moxifloxacin and liposomal amphotericin B were started and, because of the persistence of neutropenia (grade IV), the G-CSF dose was doubled.

**Figure 2:**
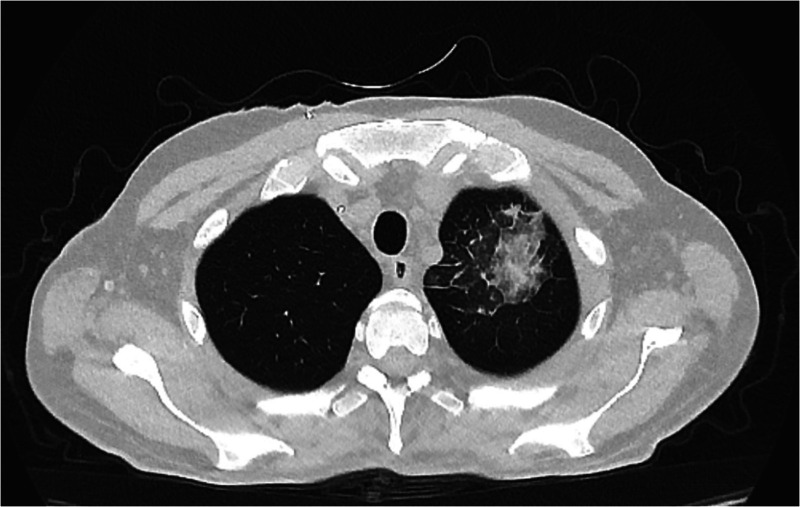
Computed tomography of the chest (axial image).

**Figure 3:**
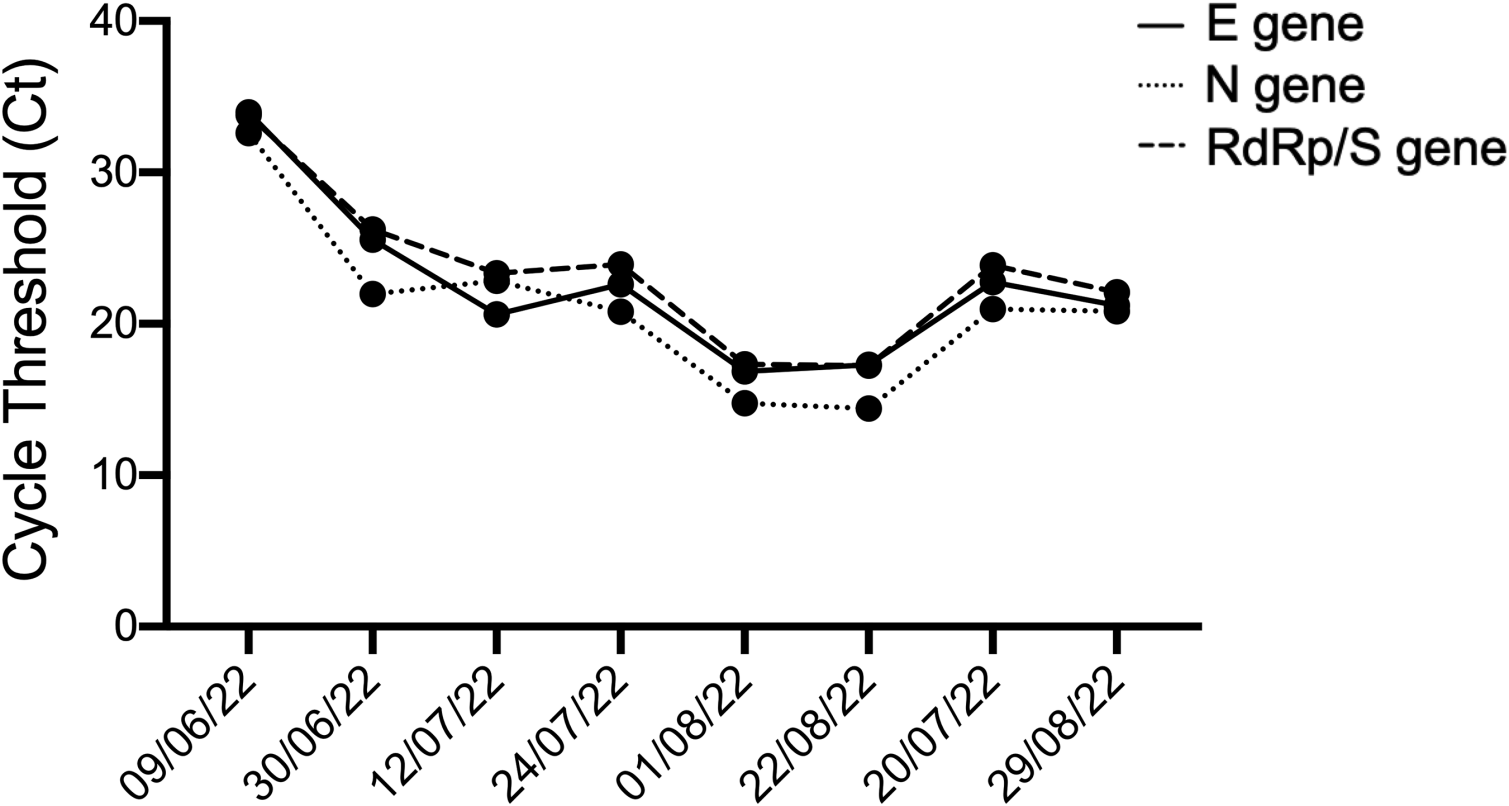
Cycle threshold (Ct) values for the E, N, and RdRP/S genes at different time intervals, before and after CAR-T therapy, to determine dynamics of the SARS-CoV-2 viral load. Each dot represents a different time point.

On day +28, a CT scan (Figure 2) showed a worsening of pulmonary infection and the patient required high-flow oxygen therapy. On day +30 a bronchoalveolar lavage (BAL) provided the presence of *S. maltophilia*, multisensitive *Klebsiella pneumoniae*, and, unexpectedly, of the SARS-CoV-2 omicron variant. Accordingly, SARS-CoV-2 pneumonia was treated with Remdesivir (200 mg IV on day 1, then 100 mg IV on days 2–5) and liposomal B amphotericine was replaced with isavuconazole, since the *Aspergillus* positivity (BAL galattomannan positivity was 1.23) was emerging under the previous antifungal therapy. Therefore, clinical findings were compatible with a diagnosis of proven COVID-19 associated pulmonary aspergillosis (CAPA) (5). Quickly, the patient became afebrile and progressively reduced his oxygen requirement. A short course of dexamethasone (10 mg four times a day) was given as an effort to lower the signs of inflammation (ferritin >5,000 μg/L, IL-6 > 3,000 ng/L). Then, a rapid decrease in inflammatory status, a reduced oxygen need, and an increased neutrophil count were observed. Figure 3 shows the cycle-threshold (Ct) evolution for the different genes (gene E, N RdRp, and S) over the time span between lymphodepletion and after the CAR-T cells. Here, it is possible to compare the different Cts for the different genes detected by the kit (E, N RdRp, and S genes). All samples analyzed have Ct < 35, so there are no weakly positive samples, as 35 is the cutoff used to distinguish samples from positive to weakly positive.

The early Immune Effector Cell-Associated Hematotoxicity (ICAHT) score (6) was grade IV and the late score grade III. For persistent cytopenia G-CSF refractory 4 weeks after CAR-T infusion, an incremental work-up was performed to define the differential diagnoses. The latter included a bone marrow aspiration and biopsy. Flow cytometric immunophenotyping excluded the presence of lymphoma cells, and showed an increased presence of reactive lymphocytes, probably secondary to virosis. Between day +30 and day +90, the patient remained severely lymphopenic (<200 cells/µl), with circulating CAR-T cells progressively decreasing to less than 3% from day +90 onward.

**Figure 4:**
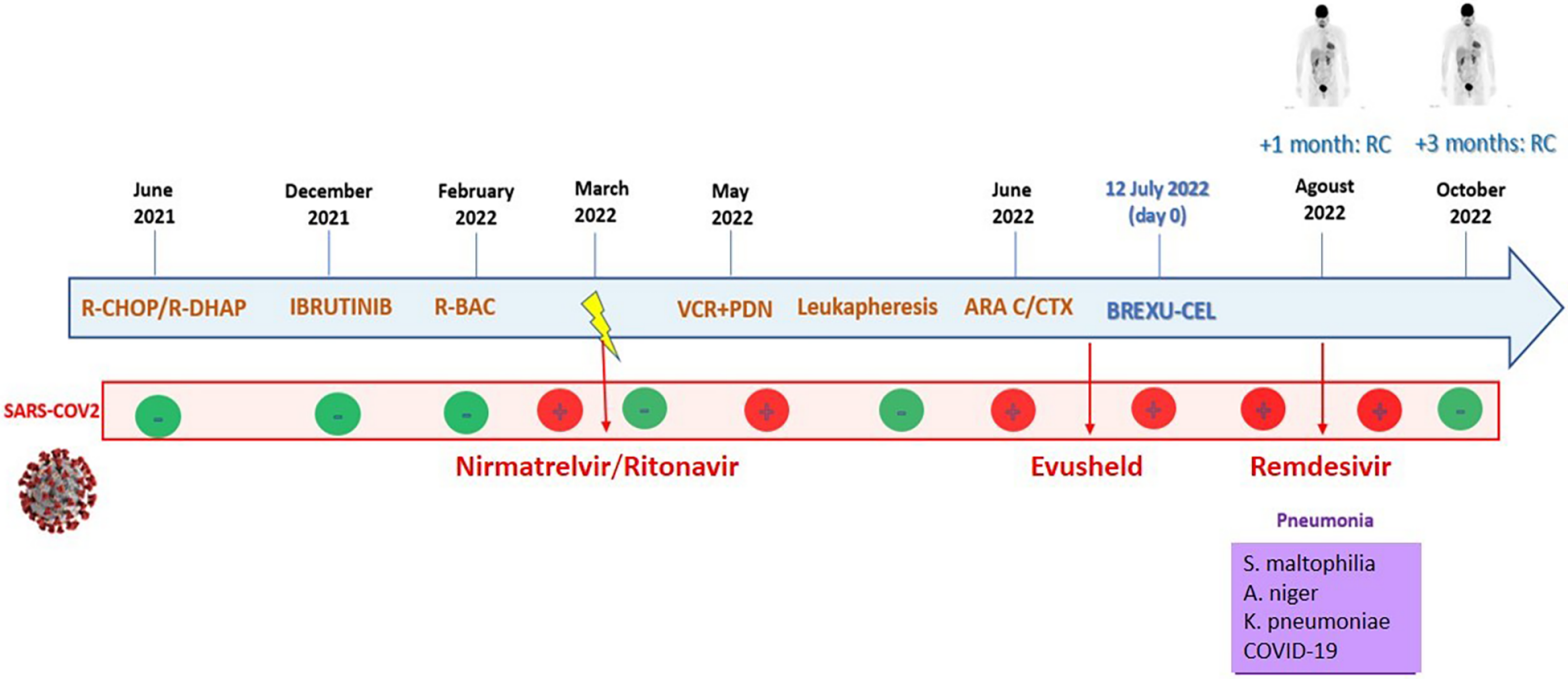
The patient's timeline. Inside the blue arrow, the main lines of therapy with the corresponding dates are reported. The second red line shows samples of SARS-CoV-2 PCR nasal swabs. Then, in the violet box, the infectious complications during CAR-T cell hospitalization are reported.

A positron emission tomography (PET) scan performed at day +42 showed complete metabolic remission (CR). Thus the patient was finally discharged in CR and proper condition on day +45 after CAR-T cell infusion. At day +120, he is well, still in complete remission, and COVID free (Figure 4).

## Material and methods

### Detection of SARs-CoV-2 and variant typing

Nasopharyngeal specimens were collected at the Brescia Civic Hospital, (Brescia, Lombardy, Italy), using FLOQSwabs in the universal transport medium (UTM) (COPAN, Brescia, Italy). Viral RNA was extracted from 300 µl of UTM with Nimbus automatic system (Arrow Diagnostics, Genoa, Italy), according to the manufacturer's instructions. Amplification was performed on BioRad CFX PCR machine (Bio-Rad Laboratories S.r.l., Milan, Italy) using the Allplex™ SARS-CoV-2 Assay (Seegene Inc., Seoul, Korea), a multiplex real-time RT-PCR assay that enables simultaneous amplification and detection of four target genes of SARS-CoV-2 (E, RdRP, S, and N genes) with internal control (IC) to monitor the whole process of nucleic acid extraction and amplification. Ct values were automatically calculated using the Seegene Viewer analysis software (Seegene Inc., Seoul, Korea). A plot of cycle threshold (Ct) values was performed using Prism 8 software (GraphPad, San Diego, CA, USA). SARS-CoV-2 variant typing was performed on extracted RNA using the Allplex™ SARS-CoV-2 Variants II Assay, which detects the presence of the K417N mutation in the S gene.

### Viral RNA extraction and Sanger sequencing

Total RNA was extracted from 200 µl of nasopharyngeal swabs using a QIAamp DSP Virus Kit® (Qiagen, Hilden, Germany) according to the manufacturer's instructions. RNA was eluted in 30 µl AVE and stored at −80 °C until use. SARS-CoV-2 RNA was reverse-transcribed and PCR amplified using a SuperScript™ IV One-Step RT-PCR System with Platinum™ Taq DNA Polymerase (Thermo Fisher Scientific, Carlsbad, CA, USA) in a 50 µl reaction. PCR primers used in the reaction were: SARS2-S-F3, 5′-TAT CTT GGC AAA CCA CGC GAA CAA and SARS2-S-R3, 5′-ACC CTT GGA GAG TGC TAG TTG CCA TCT C. Then, PCR products were checked on a 1% agarose gel, purified through QIAquick PCR Purification Kit® (Qiagen, Hilden, Germany), and quantified using the Qubit DNA HS Assay Kit (Thermo Fisher Scientific). The purified PCR products were sequenced with the BigDye Terminator v3.1 cycle sequencing kit on SeqStudio Genetic Analyzer (Thermo Fisher Scientific). The derived sequences were analyzed with Geneious software (v. 11.1.5) (Biomatters Ltd., Auckland, New Zealand), using the sequence NC_045512.2 as a SARS-CoV-2 reference.

## Discussion

The infection of immunocompromised individuals with SARS-CoV-2 presents distinct challenges, marked by prolonged viral shedding and compromised inflammatory responses. This phenomenon has been observed in our patient who, due to their immunocompromised state as a result of ongoing chemotherapy, exhibited protracted viral shedding without experiencing symptoms or virus-related complications (7–11). This unique case prompted a complex decision-making process involving the CAR-T Team physicians and the patient.

Previous studies have highlighted the poor outcomes of COVID-19 in immunocompromised patients, particularly in the context of CAR-T recipients, where mortality rates can escalate up to 40% (12). This raised concerns about initiating CAR-T therapy in a SARS-CoV-2 positive recipient due to the potential for disease progression, pneumonia, and the development of superinfections, such as CAPA (5).

A notable aspect of this case report is its novelty, demonstrating the feasibility of CAR-T therapy in a patient with a concurrent SARS-CoV-2 infection. While the decision was intricate, the patient's positivity for the virus did not preclude the pursuit of CAR-T therapy, especially considering the absence of pulmonary involvement.

The comprehensive management employed for this patient's case played a crucial role in the positive outcome. Swift initiation of tocilizumab, combined with aggressive antibacterial, antifungal, and anti-COVID treatments alongside high-flow oxygen support, effectively resolved CRS and CAPA. In addition, the use of dexamethasone proved instrumental in controlling inflammation and promoting hematologic recovery, mitigating the risks associated with pancytopenia.

Looking ahead, the management of SARS-CoV-2 positive recipients who are candidates for allogeneic hematopoietic stem cell transplantation or CAR-T therapy necessitates careful consideration of evolving treatment strategies (13–16). Despite the complexities, the successful outcome for our patient underscores the potential for a multidisciplinary approach and underscores the importance of individualized patient care in such intricate cases.

In conclusion, this case report offers valuable insights into the feasibility of CAR-T therapy for SARS-CoV-2 positive immunocompromised patients. It underscores the significance of early intervention, comprehensive management, and collaborative decision-making in achieving positive outcomes for patients who face dual challenges of immunosuppression and COVID-19 infection. Furthermore, it points toward the potential of emerging treatment strategies in guiding the management of SARS-CoV-2 positive recipients in the realm of cellular therapy.

## Conclusion

In summary, the decision to administer CAR-T cells to this high-risk patient proved to be a winning strategy. In our case, despite the presence of multiple infections, a complete remission of the lymphoma was obtained, secondary to a good expansion of CAR-T cells, which is consistent with the conclusions of several studies, showing a strong correlation between CAR-T cell expansion and disease outcome (17–19). Indeed, the patient at day +120 is well, in complete remission, and COVID free (Figure 4).

## Data Availability

The original contributions presented in the study are included in the article/Supplementary Material, further inquiries can be directed to the corresponding author.
